# Evaluation of NMU-Induced Breast Cancer Treated with Sirolimus and Sunitinib on Breast Cancer Growth

**DOI:** 10.31557/APJCP.2020.21.10.2919

**Published:** 2020-10

**Authors:** Nurul Fathiyatul Nabila Jaffar, Muhammad Shahidan Muhammad Sakri, Hasnan Jaafar, Wan Faiziah Wan Abdul Rahman, Tengku Ahmad Damitri Al-Astani Tengku Din

**Affiliations:** 1 *Department of Pathology, School of Medical Sciences, Universiti Sains Malaysia, Kubang Kerian, Malaysia.*; 2 *Department of Chemical Pathology, School of Medical Sciences, Universiti Sains Malaysia, Kubang Kerian, Malaysia. *

**Keywords:** Sirolimus, sunitinib, estrogen receptor, progesterone receptor, human epidermal growth factor receptor-2

## Abstract

**Objective:**

To analyze the effect of sirolimus and sunitinib in blocking the tumor growth and to evaluate the expressions of estrogen receptor (*ER*), progesterone receptor (*PgR*), and human epidermal growth factor receptor-2 (HER2/neu) after treated with sirolimus and sunitinib.

**Methods:**

Thirty-two female Sprague Dawley rats at age 21-days old were administered intraperitoneally with N-Methyl-N-Nitroso Urea (NMU), dosed at 70mg/kg body weight. The rats were divided into 4 groups; Group 1 (Control, n=8), Group 2 (Sirolimus, n=8), Group 3 (Sunitinib, n=8) and Group 4 (Sirolimus+Sunitinib, n=8), being treated twice when the tumor reached the size of 14.5±0.5 mm and subsequently sacrificed after 5 days. The protein expressions of *ER, PgR* and *HER2/neu* of the tumor tissues were evaluated by using immunohistochemistry analysis.

**Results:**

Treatment with sirolimus alone lowered expressions of *ER* and *PgR* of breast cancer and reduced tumor size. There was no significant difference of *ER* and *PgR* expressions between control and sunitinib treated tumor. Sunitinib treated tumors reduce in diameter after the first treatment, however the diameter increases after the second treatment. Histologically, sunitinib treated tumor did not show any aggressive invasive carcinoma of no special type (NST) histological subtypes. In addition, all NMU-induced tumors are HER2/neu-negative scoring.

**Conclusion:**

Sirolimus is neither synergistic nor additive with sunitinib for breast cancer treatment.

## Introduction

Breast cancer is a heterogeneous disease with a wide variety of clinical, pathological, and molecular characteristics, the most commonly diagnosed cancer among females and the leading cause of women cancer death (Bray et al., 2018). According to the Malaysia National Cancer Registry Report (2019), breast cancer accounted for 34.1% of all female cancer cases. Breast cancer is the leading cause of cancer death in Malaysia with total of 21,634 cases of female breast cancer was diagnosed for the period of 2012-2016 (Azizah, 2019). Hence, it is compulsory to conduct research to understand the pathogenesis of breast cancer and discover the targeted therapy for the detection and treatment of breast cancer. 

Estrogen hormone is important in normal mammary cells to regulate growth, differentiation and maintain homeostasis. Estrogen can acts as a mitogen that stimulates breast tissue to mitosis, and certain metabolites of estrogen also can act as carcinogens or genotoxin that directly damaging DNA, thereby causing cancer cells to form (Cavalieri and Rogan, 2011). However, the effects of estrogen hormone alone do not fully lead to breast cancer development. Breast cancer tumors are dependent on estrogen and/or progesterone hormones for growth and this effect is mediated through estrogen receptor (ER) and progesterone receptor (PgR). Human epidermal growth factor receptor-2 (HER2/neu) belongs to a family of four homologous receptors involved in the tyrosine kinase mediated regulation of normal breast tissue growth and development (Iqbal and Iqbal, 2014). The high expression of *HER2/neu* in breast cancer results indicates of more aggressive tumor with a poor prognosis. Hormone receptor studies such ER, PgR, and HER2/neu are routinely done in breast carcinoma. It not only helps in the prognosis of the tumor but also helps in deciding the best treatment.

In order to understand the biology of cancer and develop cancer prevention strategies, chemically induced carcinogenesis models in rat are widely used. There are several types of carcinogen used to induce cancer in an animal model such as 1,2 dimethylbenz (a)-anthracene(DMBA), Diethylnitrosoamine (DEN), Azoxymethane (AOM), and N-Nitroso-N-Methylurea (NMU). NMU is administrated intraperitoneally to animals to induce the oncogenesis of the mammary ducts and yields a high incidence of estrogen and/or progesterone receptor (ER/PgR)-positive mammary tumors. NMU-induced mammary carcinoma is age dependent; and the model is widely used to screen and evaluate the potency of cancer-suppressing and promoting agents.

Sirolimus, also known as Rapamycin is a natural macrocyclic lactone produced by the bacterium Streptomyces hygroscopicus, with immunosuppressant properties (Martel et al., 1977). It was initially developed as an antifungal agent until it was discovered to have effective immunosuppressive and anti-proliferative properties due to its ability to inhibit mammalian target of rapamycin (mTOR) (Li et al., 2014). Sirolimus is a mammalian target of rapamycin inhibitor that has been shown to inhibit rather than promote cancers in experimental models. Sirolimus target mTORC1. Blocking of mTORC1 will inhibit cell growth factors, nutrients, energy and oxygen status supply that are required for cell growth and proliferation. However, long-term exposure to sirolimus will inhibit mTORC2 by isolating newly synthesized mTOR molecules (Guduru and Arya, 2017).

Sunitinib (Sutent) is a tyrosine kinase inhibitor (TKI) indicated for first-generation multi-targeted ATP-competitive TKIs including the vascular endothelial growth factor receptors (VEGFRs) types 1 and 2 (FLT1 and FLK1/KDR), the platelet-derived growth factor receptors (PDGFR-α and PDGFR-β), the Fms Related Receptor Tyrosine Kinase (FLT3), Rearranged during Transfection (RET) kinases, and the stem cell factor receptor c-Kit. The VEGF family are frequently overexpressed in various solid tumors including mammary tumor and bind to vascular endothelium to induce angiogenesis. Inhibiting these tyrosine kinase receptors will block downstream signal transduction, thus affecting tumor angiogenesis and growth. Its antiangiogenic properties are well established and approved by Food and Drug Administration (FDA) in the use against treatment of gastrointestinal stromal tumor (GIST) after disease progression on or in tolerance to imatinib mesylate (Lopes and Bacchi, 2010), advanced renal cell carcinoma (RCC) (Adams and Leggas, 2007), adjuvant treatment of adult patients at high risk of recurrent RCC following nephrectomy (Fadil Hassan, 2018), pancreatic neuroendocrine tumors (pNET) in patients with not resectable locally advanced or metastatic disease (Delbaldo, Faivre, Dreyer, and Raymond, 2012) and hepatocellular carcinoma (HCC) (Zhu et al., 2009). 

On the basis of these data, we therefore sought to explore the effects of sirolimus and sunitinib treated of NMU induced tumors on the tumor growth, histological changes, and protein expressions of *ER, PgR *and *HER2*.

## Materials and Methods

*Animal procedures*

Thirty-two female of Sprague Dawley rats were acquired from the Animal Research and Service Centre (ARASC), USM. The rats were then caged in environmentally controlled conditions (temperature 23 ± 2°C, relative humidity 70 ± 5%, and alternate 14 h light 10 h dark cycle). They were fed with food pellets and tap water ad libitum. The principles in the care and use of laboratory animals were strictly in accordance with USM animal ethics guidelines and with supervision and husbandry facilities provided by ARASC (USM/IACUC/2017/(108)(876).

*NMU, Sirolimus and Sunitinib preparation*

N-Methyl-N-Nitroso Urea (Cat. No. M325815) was provided by Toronto Research Chemicals, Canada. The off-white wet solid of NMU was freshly-prepared prior to injection by dissolved in 70 mg/kg body weight NMU with 0.9% normal saline followed by gentle heating in water bath and vigorous shaking. Both sirolimus and sunitinib were prepared by dissolving the powder in 10% DMSO, 40% PEG300, 5% PEG (80) and 0.9% normal saline to make up 1 ml solution to a final concentration of 10 mg/0.2ml, while Sunitinib was dissolved in normal saline to the final concentration of 5 mg/0.2ml. 

*Tumor Induction and Detection*

The NMU at a dose of 70 mg/kg body weight was injected intraperitoneally two times. The first NMU injection was administrated when the rat’s age were 21 days old, followed by second injection at the alternate days. The rats were weighed and palpated twice a week for the detection of mammary tumors. The mammary lesions growths were observed and their diameter size was measured by using Vernier calliper. The symptoms of illness or side effects which may cause by NMU toxicity were also observed.

*Experimental design*

All rats were randomly assigned into four groups. Group 1 (n=8) served as a control group and was sacrificed after 5 days injection with physiological normal saline (used as a placebo) at size of 14.5 ± 0.5 mm. For the treated groups, the rats were anesthetized by Ketamine/Xylazine. Then, the rats in Group 2 (n=8) were treated with sirolimus, Group 3 (n=8) with sunitinib, and Group 4 (n=8) with sirolimus + sunitinib via an intralesional injection when the lesions reached diameter size of 14.5±0.5 mm twice for alternate days. The solution was freshly prepared prior to injection and kept on ice until intervention process. The rats in Group 2, 3 and 4 were sacrificed when the lesions regressed post 5 days intervention.

*Tumor sample collection*

All tumor-bearing rats were sacrificed by euthanized through exposure to carbon dioxide gaseous in a closed chamber. At necropsy, all grossly visible mammary tumors and normal breast pad were removed. A portion of each sample was fixed at room temperature in 10% normal buffered formalin while the remaining was fixed in RNA later solution. Tumor tissues were fixed in 10% normal buffered formalin for 24 hours at room temperature to obtain adequate fixation and optimal histology outcomes. The tissues were processed and embedded in paraffin for routine histological evaluation. Then, the paraffin sections of all tissues excised at necropsy were stained with Hematoxylin and Eosin (H and E).

*Immunohistochemistry*

Briefly, sections were deparaffinized, hydrated, and blocked for endogenous peroxidase. Heat-induced epitope retrieval was performed for all antibodies. Detailed staining protocols for all antibodies are listed in Table 1. 

For ER and PgR protein, the nucleus staining of epithelial tumor tissues were evaluated using Allred scoring method. As per the Allred score for ER and PgR nuclear positivity, the proportion score (PS) (0–5) and the % positive tumor cells are respectively, 0 (0%), 1 (<1%), 2 (1–10%), 3 (11–33%), 4 (34–66%), and 5 (67–100%).The intensity of staining (IS) for the nuclear positivity of the cells graded as 0, 1, 2, and 3 was as none, mild, moderate, and strong, respectively. The total scores for ER and PgR are given as TS = PS + IS. TS 0 and 2 are negative scores, and 3, 4, 5, 6, 7, and 8 are positive scores. 

Membrane and/or cytoplasmic staining for HER2/neu antigen were scored using American Society of Clinical Oncology (ASCO)/College of American Pathologists (CAP) guideline. HER2/neu test result was positive for HER2/neu 3+ based on circumferential membrane staining that is complete, intense. HER2/neu 2+ was equivocal based on circumferential membrane staining that is incomplete and/or weak/moderate and within >10% of the invasive tumor cells; or complete and circumferential membrane staining that is intense and within ≤10% of the invasive tumor cells. HER2/neu test result is negative if; IHC 1+ as defined by incomplete membrane staining that is faint/barely perceptible and within >10% of the invasive tumor cells and; IHC 0 as defined by no staining observed or membrane staining that is incomplete and is faint/barely perceptible and within ≤10% of the invasive tumor cells.

*Statistical analysis*

Data was descriptively presented in mean ± S.D or median ± IQR and frequency (percentage). The differences in terms of immunohistochemistry expressions across all experimental groups were determined by the Kruskal–Wallis test with Bonferroni correction followed by Pairwise test for multiple comparisons. All statistical analyses were carried out using Statistical Product and Service Solutions (SPSS) version 26 (IBM, New York USA).

## Results

*Tumor incidence and latency*

The sums of mammary tumor which were successfully excised from the rats were 35 tumors. Among them, 19 tumors (54.3%) were observed to be located in the abdominal inguinal region while 16 tumors (45.7%) located in cervical thoracic region of mammary gland chain. At macroscopic, they were solid and receiving blood supply from collateral vessels.

*Tumor diameter progression*

The tumor diameter in sunitinib treated group regressed after first injection to 12.5 ± 2.2 mm. However, five days after second treatment intervention, the tumor diameter increased to 14.2 ± 2.5 mm. In sirolimus treated group, the tumor diameter significantly regressed after treatment to 6.3 ± 3.0mm. In sirolimus + sunitinib treated group, the tumor diameter also regressed to 11.2 ± 2.4 mm after five days intervention of second treatment (Table 2).

*Histological Analysis*

**Figure 1:**
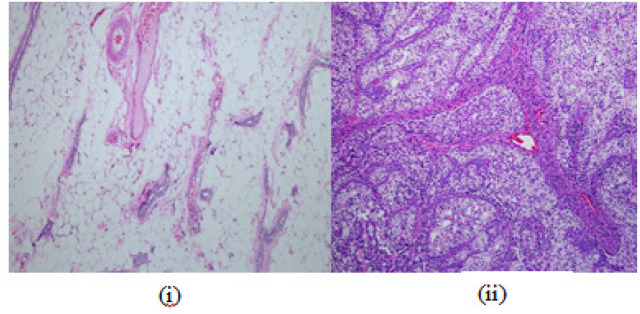
Comparison of (i) Normal mammary gland of female Sprague-Dawley rat, and (ii) NMU-induced Invasive Breast Carcinoma. H&E staining magnification 100X

**Figure 2:**
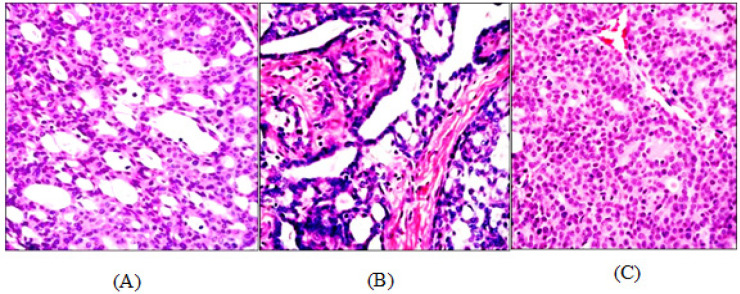
(A) NMU-induced Invasive Carcinoma of Cribriform. Characterized by well-defined spaces formed by arches of cells, tumor cells are small, moderate nuclear pleomorphism with no / rare mitotic figures. (B) NMU-induced Invasive Carcinoma of Papillary. The tumor has numerous papillary projections with thin fibrovascular core. (C) NMU-induced Invasive Carcinoma of No Special Type. The carcinoma displayed diffuses infiltration of neoplastic cells, with less tubule formation, highly nuclear pleomorphism and high mitotic rate. H&E staining magnification 400x

In control group, a total of 8% of NMU mammary tumors were observed to show less aggressive Invasive Breast Carcinoma (IBC) cribriform subtype whilst the remainder 92% was aggressive subtypes (IBC papillary and IBC-NST). However, no benign mammary tumor was observed. Figure 1 showed comparison of histological features of normal mammary gland with the malignant invasive carcinoma, and the histology of IBC of cribriform, papillary and NST were shown in Figure 2.

As shown in Table 3, the trend of Invasive Carcinoma pattern in tumor tissues for control group showed the highest papillary pattern (56.25%). However, the Invasive Carcinoma of Papillary pattern decreased in Sirolimus-treated group (18.75%). The Sirolimus-treated group showed the highest less aggressive Invasive Carcinoma of Cribriform pattern (56.25%). In contrast, the tumor tissues treated with sunitinib and sirolimus + sunitinib treated groups showed the highest pattern of papillary pattern (56% and 56.25% respectively) compared to sirolimus-treated group.

*Immunohistochemistry*

**Figure 3:**
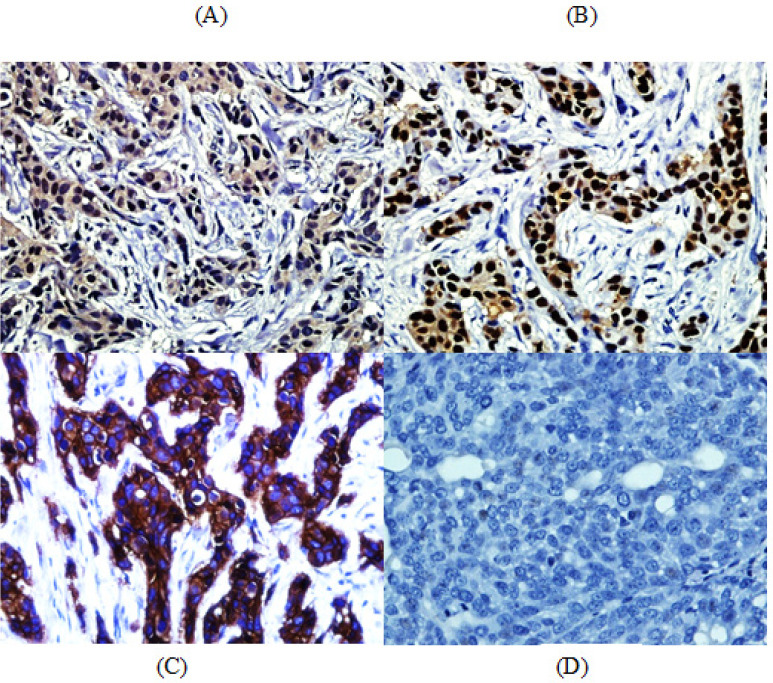
Immunohistochemistry Expressions of Representative Markers on Tumor Specimens. (A) Invasive Carcinoma positive control for ER, (B) Positive staining of PgR in Invasive Breast Carcinoma, (C) Positive control for HER2/neu, (D) Negative staining of ER in Invasive Breast Carcinoma. (Original magnification x400)

The results of protein expressions of *ER, PgR*, and *HER2/neu* on tumor are shown in Figure 3 and the IHC scoring are summarized in Table 4. The statistical analyses are shown in Table 5 and Table 6.

For Estrogen Receptor (ER), Kruskal-Wallis test provided very strong evidence of a difference (p= 0.000) between the median ranks of at least one pair of groups. Pairwise tests were carried out for the four pairs of groups. There was very strong evidence of ER total score readings between sirolimus treated and sunitinib treated group (p= 0.020, adjusted using the Bonferroni correction), sirolimus treated and control group of a difference, (p= 0.000, adjusted using the Bonferroni correction), and ER total score of control and combinational-treated group (p= 0.011, adjusted using the Bonferroni correction). The median ER total score for the control group was 6.5 compared to 4.0 in the sirolimus-treated group. There was no evidence of a difference between the other pairs.

Progesterone Receptor (PgR) Kruskal-Wallis test showed significant difference (p= 0.000) between the median ranks of at least one pair of groups. Pairwise tests were carried out for the four pairs of groups. There were significant differences in PgR total score readings between sirolimus treated and sunitinib treated group (p= 0.003, adjusted using the Bonferroni correction), sirolimus treated and control group of a difference, (p= 0.000, adjusted using the Bonferroni correction), and PgR total score of control and sirolimus + sunitinib treated group (p= 0.0038). The median PgR total score for the control group was 8.0 compared to 5.0 in the sirolimus-treated group. There was no evidence of a difference between the other pairs.

Kruskal-Wallis test for *HER2/neu* expression also provided a difference (p= 0.043) between the median ranks of at least one pair of groups. However, the pairwise tests carried out for the four pairs of groups showed no strong evidence of HER2/neu total score readings between all four groups when the p-value for all comparisons is >0.05. The median for HER2/neu total score for all groups was 1. Hence, there was no evidence of difference between all pairs.

**Table 1:** Staining Protocol of IHC

Primary antibody	Dilution	Antigen retrieval	Blocking
Estrogen receptor (ER)	1:100	Tris-EDTA (pH 9)	15 minutes
Progesterone receptor (PgR)	1:200	Citrate buffer (pH6)	15 minutes
HER2/neu	1:100	Tris-EDTA (pH 9)	15 minutes

**Table 2:** Tumor Diameter (Mean ± S.D) of Intervention Groups after First Treatment and Five Days Post Second Treatment

Groups	Tumor diameter (Mean ± S.D), mm	
	After first treatment	Five days after second treatment
Sirolimus	11.3 ± 4.0	6.3 ± 3.0
Sunitinib	12.5 ± 2.2	14.2 ± 2.5
Sirolimus + Sunitinib	12.6 ± 3.2	11.2 ± 2.4

**Table 3:** The Tumor Types in the Intervention Groups

Groups	Invasive Carcinoma		
	Cribriform (%)	Papillary (%)	No Special Type- NST (%)
Control	1/16 (6.25%)	9/16 (56.25%)	6/16 (37.5%)
Sirolimus	9/16 (56.25%)	3/16 (18.75%)	4/16 (25%)
Sunitinib	7/16 (44%)	9/16 (56%)	0/16 (0%)
Sirolimus + Sunitinib	5/16 (31.25%)	9/16 (56.25%)	2/16 (12.5%)

**Table 4:** Kruskal-Wallis Test Table for Total Score of ER, PgR, and HER2/neu

Group	ER			PgR			HER2/neu		
	Median (IQR)	Chi-square (df)	*p* value	Median (IQR)	Chi-square (df)	*p* value	Median (IQR)	Chi-square (df)	*p* value
Control	6.5 (1)	29.353 (3)	0.000*	8.0 (1)	27.426 (3)	0.000*	1.0 (1)	8.142 (3)	0.043*
Sirolimus	4.0 (1)			5.0 (2)			1.0 (1)		
Sunitinib	5.0 (1)			7.0 (1)			1.0 (1)		
Sirolimus + Sunitinib	5.0 (1)			6.0 (2)			1.0 (1)		

*significant value: p < 0.05

**Table 5:** The Expressions of ER, PgR, and HER2/neu between Intervention and Control Groups

Group	p-value		
	ER	PgR	HER2/neu
Control vs Sirolimus	0.000*	0.000*	0.446
Control vs Sunitinib	0.087	0.625	1
Control vs Sirolimus + Sunitinib	0.011*	0.038*	0.815

*, Pairwise Test; *, significant value: p < 0.05

**Table 6:** The Expressions of ER, PgR, and HER2/neu amongst the Intervention Groups

Group	p-value		
	ER	PgR	HER2/neu
Sirolimus vs Sunitinib	0.020*	0.003*	0.093
Sirolimus vs Sirolimus + Sunitinib	0.142	0.109	1
Sunitinib vs Sirolimus + Sunitinib	1	1	0.2

*, Pairwise Test; *, significant value: p < 0.05

## Discussion

In this study, we investigated the synergistic effects of sirolimus (anti-mTOR) and sunitinib (multi-targeted receptor tyrosine kinase inhibitors) on NMU-induced breast cancer. The novel findings in this study are: 1) Treatment with sirolimus alone lowered expressions of *ER* and *PgR* of breast cancer and reduced tumor size; 2) There is no significant difference in *ER* and* PgR *expressions between control tumor and Sunitinib treated tumor; 3) Sunitinib treated tumors reduce in diameter after the first treatment, but the diameter increases after the second treatment; 4) Sunitinib treated tumor does not show any aggressive invasive carcinoma of NST histological subtypes; 5) All NMU-induced tumors are HER2/neu-negative scoring. 

Breast cancer is dependent on estrogen and/or progesterone hormones for growth and this is mediated through estrogen receptors (ERs) and progesterone receptors (PgRs). ER specifically ERα contributes to tumor aggressiveness and the increasing histological grade (Muscat et al., 2013). As Booth and Smith, (2006) reported that ER and PgR are localized in the nucleus of epithelial cells and will co-upregulated to label-retaining mammary epithelial cells that divide asymmetrically and retain their template DNA strands. In this study, it was proved that anti-tumor treatment potentially preventing the estrogen dependent mechanism towards cancer cells progression and lowered the *ER* and *PgR* expression. 

Our study disclosed that all malignant breast carcinoma of control tumor tissue are both overexpressed of ER and PgR. However, the *ER* and *PgR* expressions of sirolimus-treated and sirolimus + sunitinib treated tumor are significantly lower compared to control group. In relation with tumor diameter, the sirolimus-treated and sirolimus + sunitinib treated tumor noticeably reduced the tumor size of NMU-induced breast cancer. These suggest that the treatment reduces the growth of premalignant rat mammary lesions and inhibits the malignant transformation of mammary carcinoma. 

However, sunitinib does not reduce the expressions of *ER* and *PgR* expressions. This result may be supported with previous research conducted by Miller et al., (2010) that showed direct inhibition of PI3K/mTOR pathway effectively suppressed the growth of both estrogen-independent and -dependent cells breast cancer cell growth associated with hyperactivation of the IGF IR/InsR/PI3K/mTOR pathway, but inhibition of nodes upstream (RTKs) and downstream (mTOR) of PI3K only partially blocked breast cancer cell growth. 

Sunitinib has been used as anticancer treatments in several types of tumor including breast cancer, however previous clinical observations of sunitinib treatment showed that this therapy has limited efficacy. Researchers figured out that when sunitinib as anti-angiogenic agents administered on an intermittent schedule; 4 weeks on and 2 weeks off, tumor regrowth was seen during drug-free periods (Burstein et al., 2008) or upon discontinuation of the treatment (Mulet-Margalef and Garcia-Del-Muro, 2016). This result was explained due to sunitinib inhibits primary tumor growth, but the inhibition is exceptional lasting responses and only show moderate increases in progression-free survival and little benefit in overall survival. This might relate to previous studies when sunitinib treated tumors reduce in diameter after first treatment, but the diameter increases after second treatment. Sunitinib might generate intratumoral hypoxia modulating the metastatic process (Lu and Kang, 2010) and stimulating cancer stem cells (CSC) (Seidel et al., 2010). 

HER2/neu-positive breast cancer is a more aggressive type of breast cancer compared with HER2/neu-negative types. Our study showed all NMU-induced breast cancers are estrogen and progesterone receptors positive but HER2/neu negative (all luminal A subtype). In the present in-vivo study, all induced mammary tumors in female rats were adenocarcinomas (luminal A subtype) based on the results of the ER and/or PgR positivity and HER2/neu negativity, as in previous report (Kinoshita et al., 2016). The negative expressions of *HER2/neu* in our result may explain decreasing grade in aggressiveness of invasive carcinoma of NST of treated groups. In addition, sunitinib as tyrosine kinase inhibitor might not be able to work efficiently to inhibit HER2/neu which involved in the tyrosine kinase mediated regulation of mammary gland since HER2/neu does not well express in NMU-induced breast cancer.

In conclusion, treatment with sirolimus alone showed significant mammary tumor inhibition which presumably exerts its inhibitory effect through mTOR pathway. ER and PgR play a major role in breast cancer cell development and positively correlated with breast cancer cell proliferation. *ER* and *PgR* expressions of sirolimus treated and combination treated tumor are significantly lower compared to control group, hence evidently reduced tumor size of NMU-induced breast cancer. In contrast, treatment with sunitinib shrinks the solid tumor after first treatment, but the diameter increases after second treatment. This might due to sunitinib generate intratumoral hypoxia modulating the metastatic process and stimulating cancer stem cells in NMU-induced mammary tumor growth. Thus, present results suggested that sirolimus is not synergistic or additive with sunitinib. Furthermore, sunitinib might be an antagonist towards the sirolimus activity as multi-targeted tyrosine kinase inhibitor.
